# Abnormal neonatal sodium handling in skin precedes hypertension in the SAME rat

**DOI:** 10.1007/s00424-021-02582-7

**Published:** 2021-05-24

**Authors:** Linda Mullins, Jessica Ivy, Mairi Ward, Olav Tenstad, Helge Wiig, Kento Kitada, Jon Manning, Natalia Rakova, Dominik Muller, John Mullins

**Affiliations:** 1 Molecular Physiology Laboratory, BHF Centre for Cardiovascular Science, Queen’s Medical Research Institute, University of Edinburgh, Edinburgh, UK; 2 Department of Biomedicine, University of Bergen, Bergen, Norway; 3 Department of Pharmacology, Kagawa University, Takamatsu, Japan; 4 EMBL-EBI, Wellcome Genome Campus, Hinxton, UK; 5 Experimental and Clinical Research Center, a joint cooperation of Max Delbrück Center for Molecular Medicine and Charité-Universitäts-Medizin Berlin, Berlin, Germany; 6 Max Delbrück Center for Molecular Medicine in the Helmholtz Association, Berlin, Germany

**Keywords:** Hsd11b2, Knockout, Hypertension, Newborn, Neonatal, Rat, Salt-sensitive, Skin

## Abstract

We discovered high Na^+^ and water content in the skin of newborn Sprague–Dawley rats, which reduced ~ 2.5-fold by 7 days of age, indicating rapid changes in extracellular volume (ECV). Equivalent changes in ECV post birth were also observed in C57Bl/6 J mice, with a fourfold reduction over 7 days, to approximately adult levels. This established the generality of increased ECV at birth. We investigated early sodium and water handling in neonates from a second rat strain, Fischer, and an Hsd11b2-knockout rat modelling the syndrome of apparent mineralocorticoid excess (SAME). Despite Hsd11b2^−/−^ animals exhibiting lower skin Na^+^ and water levels than controls at birth, they retained ~ 30% higher Na^+^ content in their pelts at the expense of K^+^ thereafter. Hsd11b2^−/−^ neonates exhibited incipient hypokalaemia from 15 days of age and became increasingly polydipsic and polyuric from weaning. As with adults, they excreted a high proportion of ingested Na^+^ through the kidney, (56.15 ± 8.21% versus control 34.15 ± 8.23%; n = 4; P < 0.0001), suggesting that changes in nephron electrolyte transporters identified in adults, by RNA-seq analysis, occur by 4 weeks of age. Our data reveal that Na^+^ imbalance in the Hsd11b2^−/−^ neonate leads to excess Na^+^ storage in skin and incipient hypokalaemia, which, together with increased, glucocorticoid-induced Na^+^ uptake in the kidney, then contribute to progressive, volume contracted, salt-sensitive hypertension. Skin Na^+^ plays an important role in the development of SAME but, equally, may play a key physiological role at birth, supporting post-natal growth, as an innate barrier to infection or as a rudimentary kidney.

## Introduction

Increased sodium and water storage in skin (sub-clinical oedema) has been associated with aging and hypertension in humans [41]. However, it has not been investigated in very young or premature babies where it may impact on trans-epidermal water loss (TEWL) [27].

In adult rats the skin is recognized as a key organ for the sequestration of excess Na^+^, which may be stored with water [47] or osmotically inactive (free from water) and complexed with proteoglycans [50], and is involved in the maintenance of ECV and blood pressure homeostasis [49]. Natriuretic control of ECV on chronic exposure to high salt may also be coupled with metabolism-driven urine concentration, involving concerted urea production by the liver and muscle, and urea recycling by the kidney, as a means to conserve body water [21]. Multiple additional pleiotropic systems, including the renin–angiotensin–aldosterone system, sodium transport in the nephron, autonomic nervous control, and steroid metabolism, all work in concert with appropriate adjustments to the vasculature and baroreceptors, to control extracellular volume (ECV) and maintain blood pressure [17].

Sensitivity to salt increases cardiovascular risk in both normotensive and hypertensive cohorts [55] — a problem given the high salt intake in the Western diet — though the underlying aetiology of salt-sensitivity is unclear [11]. Salt-sensitivity is thought to reflect altered pressure-natriuresis or up-regulated Na^+^ transport, and subsequent defects in salt storage or kidney dysfunction may each play a critical role [35].

Hypertension may be irreversibly fixed or programmed early in development, and young rats are ideally sized (~ 30 g) to facilitate chronic measurements of blood pressure, metabolic status, and kidney function, making it possible to discern which homeostatic control systems are causal and which effect the progression of hypertension. Critical early developmental stages include foetal development in utero, birth, suckling, and the introduction to solid food at weaning. At birth, the neonate experiences a brief surge in glucocorticoids, which stimulates lung development. Glucocorticoid levels then fall sharply and remain low until about 14 days of age, when they climb again, peaking at about 21 days [36]. The glucocorticoid surge parallels an increase in the potassium channel, ROMK [15, 58]. Urine concentrating capacity is absent at birth but builds slowly, as water excretion capability improves with increasing vasopressin and aquaporin 2 (Aqp2) production [7]. Nephrogenesis continues beyond birth, with nephrons not reaching maturity until about P14. An additional stage when Na^+^ handling could be critical is when the neonate begins to eat solid food during the third week of age, which is paralleled by the glucocorticoid surge. Potassium status is inversely related to blood pressure in both human and experimental models, since K^+^ is depleted when Na^+^ is retained in the kidney [22]. For example, adrenocorticotropic hormone treatment causes substantial hypokalaemia, [2, 10] which is often associated with polydipsia and polyuria [3].

Genetic modification of key genes implicated in blood pressure control or salt handling provides useful animal models to investigate salt-sensitivity. For example, loss of Hsd11b2 activity causes Na^+^ retention in principal cells of the collecting duct of the kidney, hypokalaemia, polydipsia, polyuria, volume contraction, and salt-sensitive hypertension [30]. We report that newborn rodents have very high Na and water content in their skin and use the rat SAME model to investigate neonatal sodium handling in relation to salt sensitivity.

## Results

### Electrolyte storage in skin/pelts

**Fig. 1:**
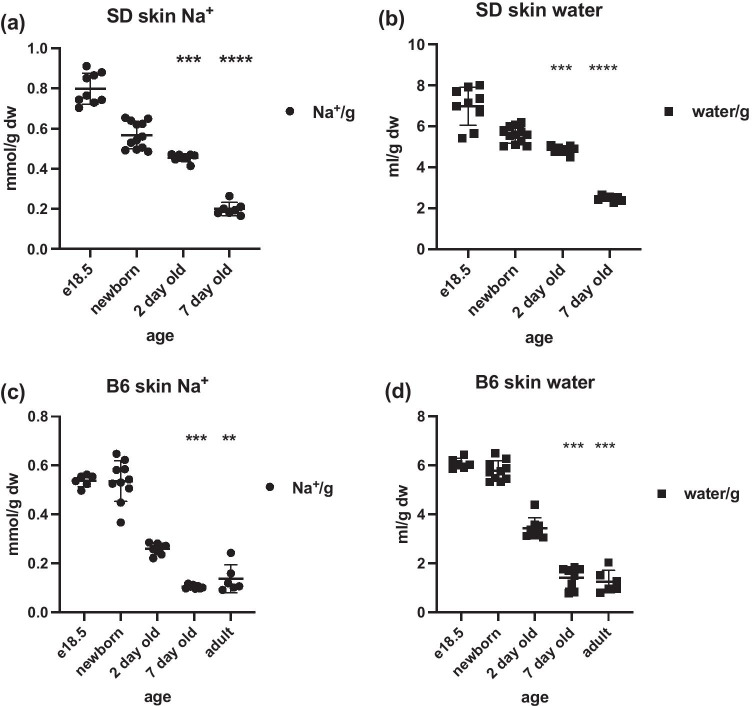
Skin Na^+^ and water content during development in Sprague–Dawley (SD) (**a**) skin Na^+^ (mmol/g dry weight; *** = 0.0002; **** < 0.0001) (**b**) skin water (ml/g dry weight; *** = 0.0009; **** < 0.0001), and C57Bl/6J mice (**c**) skin Na^+^ (mmol/g dry weight; *** = 0.0003; ** = 0.0047) and (**d**) skin water (ml/g dry weight; *** < 0.0002) (n = 6–9 per group; two-way ANOVA with Kruskal Wallis and Dunn’s multiple comparison test)

To investigate electrolyte and water content in very young rats, skin from Sprague–Dawley e18.5 embryos and neonates were analysed (Fig. 1a and b). High Na^+^ and water content was observed in e18.5 and newborn pups, which reduced significantly by 2 days of age, decreasing 2.5-fold by 7 days of age. To ascertain the generality of this observation skin samples from a separate species, C57Bl/6 J mice were collected at time points pre- and post-birth and were processed for electrolyte analysis. Again, C57Bl/6 J mouse embryos at e18.5 and birth demonstrated high levels of skin Na^+^ and water (per g dry weight), which declined rapidly in neonates, reaching a significant fourfold decline by 7 days of age (Fig. 1c–d).

**Fig. 2:**
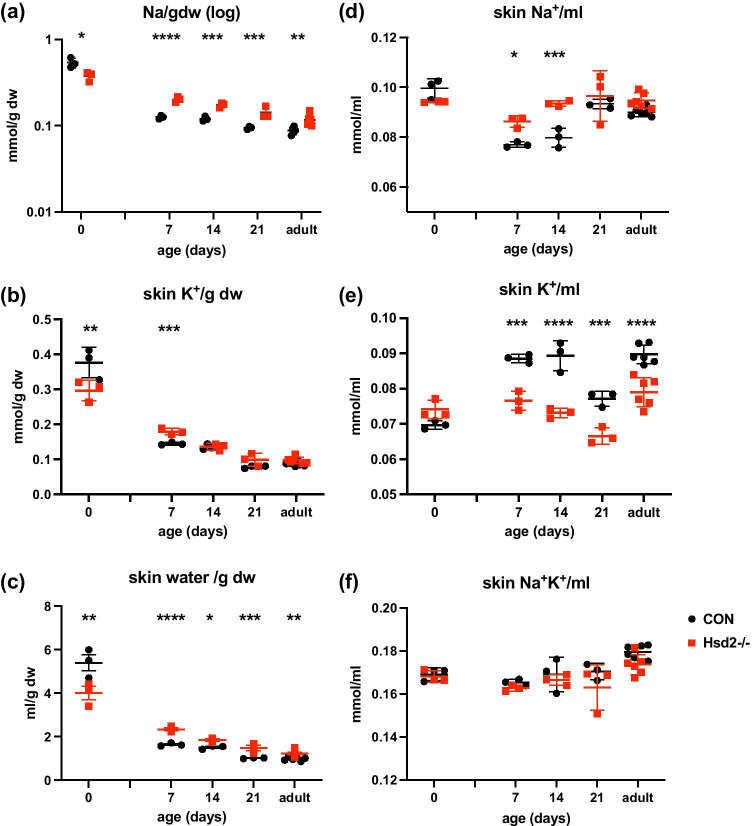
Skin electrolytes and water content during development. **a** skin Na^+^ (mmol/g dry weight; note logarithmic scale); **b** skin K^+^ (mmol/g dry weight; **c** skin water (ml/g dry weight); **d** skin Na^+^ (mmol/ml); **e** skin K^+^ (mmol/ml); **f** skin electrolytes (Na^+^K^+^(mmol/ml)). (n = 3–6 per group per time point; two-way ANOVA with Sidak’s multiple comparison test * < 0.05; ** < 0.01; *** < 0.001)

We next extended our analyses to look at a second rat strain, Fischer (F344), together with the genetically modified Hsd11b2 knockout (Hsd2^−/−^; on the same genetic background), which exhibits salt-sensitive hypertension. Fischer (F344) neonatal skin analyses again revealed dynamic changes in electrolyte and water content. Newborn control skin samples (between 6 and 18 h old) contained high quantities of Na^+^ (0.538 ± 0.071 mmol/g dry weight) and water (5.40 ± 0.66 ml/g dry weight), both of which declined approximately fourfold, towards adult levels over the first week of life (Fig. 2a–b; 7 day: Na^+^ 0.126 ± 0.006 mmol/g dry weight; water 1.64 ± 0.07 ml/g dry weight). The Hsd2^−/−^ animals, however, contained less Na + and water in newborn skin, showed a reduced rate of decline (newborn skin Na^+ ^− 0.377 ± 0.049 falling to 0.202 ± 0.015 mmol/g dry weight by day 7; newborn skin water – 4.01 ± 0.53 falling to 2.34 ± 0.12 ml/g dry weight by day 7), and retained consistently higher Na^+^ content in their pelts than controls, extending to adulthood (Fig. 2a). The Hsd2^−/−^ adults had 32% more Na^+^ content per g dry weight than controls in pelts (Hsd2^−/−^ − 0.117 ± 0.018 versus controls – 0.089 ± 0.008 mmol/g; n = 6; P = 0.0045). They also had 16% more Na^+^ content per g dry weight in bone ash (Hsd2^−/−^ − 0.515 ± 0.028 versus controls – 0.444 ± 0.020 mmol/g; P = 0.0005) and 44% more in carcass Na^+^ content per g dry weight (Hsd2^−/−^ − 0.088 ± 0.002 versus controls – 0.061 ± 0.005); P = 2.56e^−0.6^) giving a 36% increase in total body sodium (Hsd2^−/−^ − 0.135 ± 0.008 versus controls – 0.099 ± 0.006; P = 5.08e^−0.6^). Indeed, there was a strong correlation between skin Na^+^ per g dry weight relative to other variables including total body Na^+^ per g dry weight and total body water per g dry weight in Hsd2^−/−^ animals, and interestingly, a negative correlation relative to bone Na^+^ per g dry weight in both groups (Table 1). Pelt electrolyte concentration ([Na^+^ + K^+^]; Hsd2^−/−^ 0.168 ± 0.006 versus controls 0.173 ± 0.007 mmol/ml) remained remarkably constant from birth to adult (Fig. 2f), but [Na^+^] was significantly higher in Hsd2^−/−^ neonates and [K^+^] significantly lower than controls (Fig. 2d–e).

**Table 1:** Correlation coefficients (r), coefficients of determination (r^2^) and probability (p) between variables (column 1), and skin Na^+^ per g dry weight

variables	CON			Hsd2^−/−^		
	r	r^2^	p	r	r^2^	p
Total body Na^+^ g^−1^ dry weight	0.627	0.393	ns	0.951	0.905	0.0035
Total body Na^+^ + K^+^ g^−1^ dry weight	0.439	0.193	ns	0.982	0.964	0.0005
Total body water g^−1^ dry weight	0.406	0.165	ns	0.989	0.978	0.0002
Bone Na^+^ g^−1^ dry weight	− 0.911	0.83	0.0115	− 0.865	0.748	0.026

### Phenotypic characterization of neonates

**Fig. 3:**
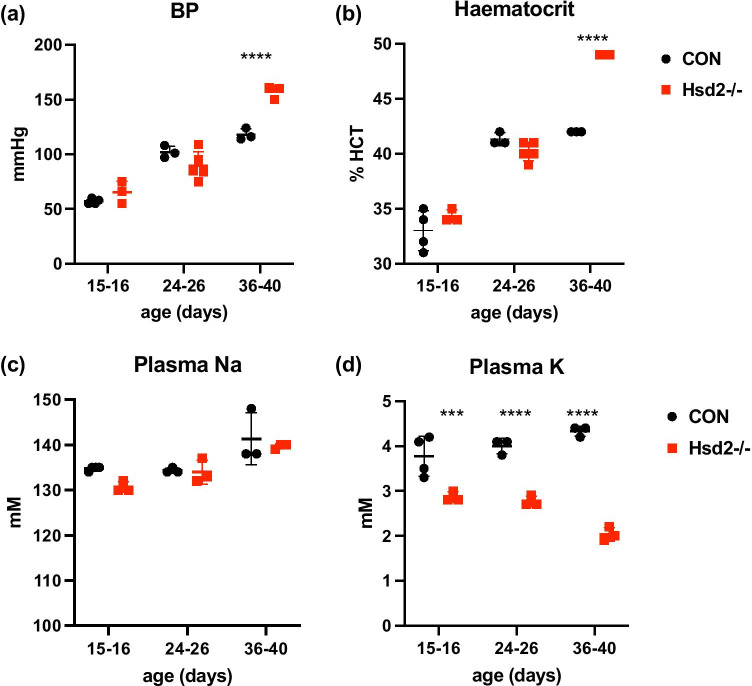
Developmental changes in **a** blood pressure **b** haematocrit **c** plasma Na^+^, and **d** plasma K^+^ (minimum n = 3 per time point; unpaired t test; *** 0.0009; **** < 0.0001)

Plasma, urine, and milk samples were collected from pups aged 13–15 days old. Hsd2^−/−^ animals already showed slight, but significant hypokalaemia at 15 days of age (Fig. 3b). Na^+^ was below detection in the urine of 13–14-day-old controls (n = 9) but was detected in 70% of age-matched Hsd2^−/−^ urine samples together with a trend towards increased K^+^ (n = 10; data not shown).

Neonatal stomach contents were analysed for electrolytes as an indirect measure of Na^+^ and K^+^ passed through mother’s milk. Since milk composition is likely to change with age of neonate, we compared electrolytes in the stomach contents at 13 days post-partum (correlation between Na^+^ and milk weight –Hsd2^−/−^: 0.963 (n = 16); controls: 0.743 (n = 10)). Samples from Hsd2^−/−^ pups contained significantly higher Na^+^ g^−1^ milk (Hsd2^−/−^ 47.36 ± 8.85 µmol/g versus control 33.57 ± 8.40 µmol/g; P = 0.0002), with a trend towards lower K^+^ (Hsd2^−/−^ 43.94 ± 9.06 µmol/g versus controls 48.52 ± 9.98 µmol/g; P = 0.238), resulting in a significantly higher Na^+^/K^+^ ratio (Hsd2^−/−^ 1.109 ± 0.264 versus 0.703 ± 0.085; P < 0.0001).

### Blood pressure, haematocrit, and plasma electrolytes

Analysis of catheterized young rats (from 15 days to 5 weeks of age; n = 3–5) suggested that MABP and haematocrit of Hsd2^−/−^ were indistinguishable from controls at 15 days old, but both reached significance by 40 days of age (MABP: 158.0 ± 6.1 mmHg versus 118.0 ± 5.3 mmHg controls; n = 3; P < 0.0001; HCT: 0.49 ± 0.01 versus 0.42 ± 0.01 controls; n = 3; P < 0.0001; Fig. 3a–b). Plasma Na^+^ (Fig. 3c) was indistinguishable from age matched F344 controls on 0.3% Na^+^ diet. However, the Hsd2^−/−^ animals showed worsening hypokalaemia, with significantly reduced plasma K^+^ (Fig. 3d) compared to F344 controls at all time points.

### Assessment of water and electrolyte balance

**Fig. 4:**
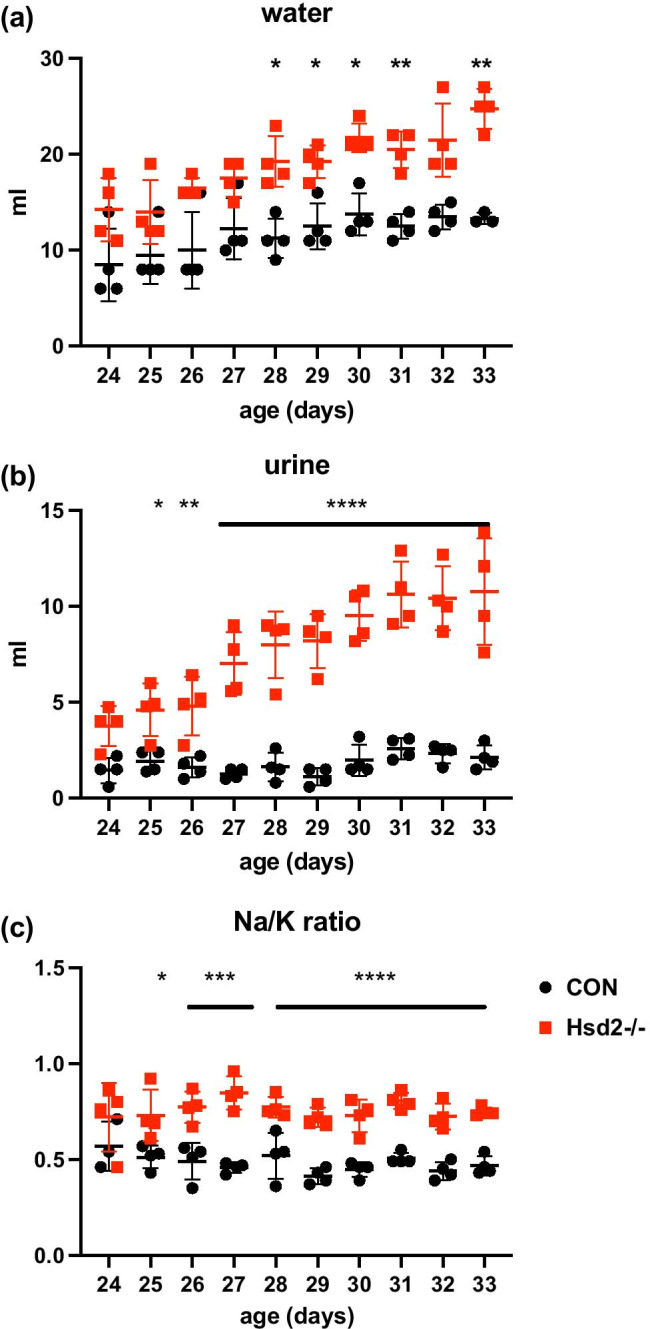
Metabolic study of post-weaned pups **a** daily water intake **b** daily urinary output **c** Na^+^/K^+^ ratio in urine (n = 4 per group; two-way ANOVA with Sidak’s multiple comparison test * < 0.05; ** < 0.01; **** < 0.0001)

Newly weaned male rats (22–23 days old; F344 versus Hsd2^−/−^; n = 4 per group) were placed in metabolic cages to determine baseline water and electrolyte balance. Hsd2^−/−^ animals developed significant polydipsia and polyuria between 23 and 33 days of age on a 0.3% Na^+^ diet (Fig. 4a–b). Young homozygotes drank significantly more water per g body weight (Hsd2^−/−^ 0.32 ± 0.03 ml versus controls 0.22 ± 0.03 ml; n = 4; P < 0.0001) and produced significantly more urine (Hsd2^−/−^ 0.15 ± 0.02 ml versus controls 0.03 ± 0.01 ml; n = 4; P < 0.0001). Hsd2^−/−^ animals had a higher urinary Na^+^/K^+^ ratio (Fig. 4c). Critically, they excreted significantly more of their ingested sodium through the kidney (Hsd2^−/−^ 56.15 ± 8.21% per g body weight compared to 34.15 ± 8.23% in controls; n = 4; P < 0.0001) from 29 days, as was observed in adult Hsd2^−/−^ animals [30].

Urinary urea concentration was significantly lower in Hsd2^−/−^ pups than controls at 32 days of age due to polyuria (196.3 ± 44.9 mM versus 746.3 ± 76.9 mM controls; n = 4; P < 0.0001), but the urea content was not significantly different (2002 ± 288 µmols versus 1710 ± 258 µmols controls; n = 4; P = 0.2469).

### Immunohistochemical analysis

**Fig. 5:**
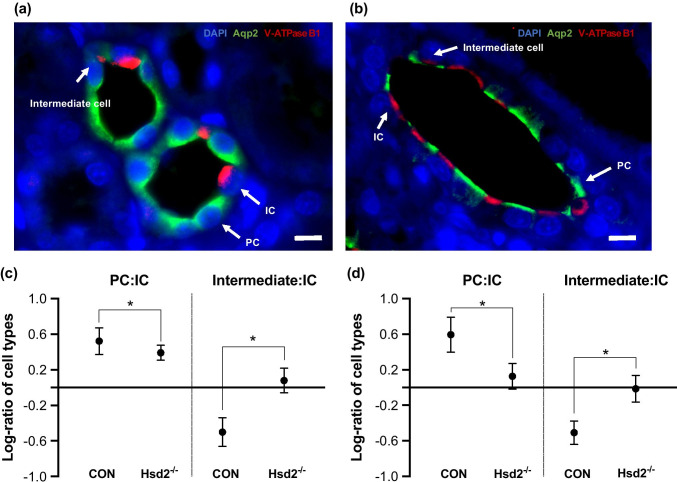
Immunohistochemical analysis showing PC, IC, and intermediate cells stained with Aqp2 and V-Atpaseb1 antibodies in **a** control; **b** Hsd2^−/−^ section. The relative proportion of cell types in collecting ducts was counted between control and Hsd2^−/−^ rats. The log-ratio of PC: IC and intermediate: IC are reported as means ± SD, at **c** ~ 5 weeks (n = 4), and **d** 24 weeks (n = 3). Significance between groups was assessed using multiple analysis of variance (p < 0.05 *). A decrease in the log-ratio of PC:IC indicates an overall decrease in the proportion of PCs present in the CD relative to ICs (the latter did not significantly change between WT and KO). Likewise, an increase in the log-ratio of intermediate: IC indicates an overall increase in the proportion of intermediate cells relative to ICs

Immunohistochemical analysis of kidney samples taken during development suggested notable changes in cell composition of the CD in Hsd2^−/−^ compared to controls (Fig. 5a–d). While no significant change in IC cell proportion in control or Hsd2^−/−^ animals was observed between ~ 5 weeks of age and 24 weeks of age, there was a significant reduction in the log-ratio of PC:IC cells (0.52 $\pm$ 0.15 control versus 0.39 $\pm$ 0.09 Hsd2^−/−^ at 5 weeks and 0.59 $\pm$ 0.20 in control versus 0.13 $\pm$ 0.14 in Hsd2^−/−^ at 24 weeks) and a significant increase in the log-ratio of intermediate: IC cells (− 0.5 $\pm$ 0.16 control versus 0.08 $\pm$ 0.14 in Hsd2^−/−^ at ~ 5 weeks and − 0.51 $\pm$ 0.13 control versus − 0.01 $\pm$ 0.15 in Hsd2^−/−^ at 24; a negative log-ratio means that the proportion of ICs is greater than that of intermediate cells) in Hsd2^−/−^ rats. This indicates that the proportion of principal cells staining positive for aquaporin decreased dramatically in Hsd2^−/−^ rats between ~ 5 weeks of age and 24 weeks of age, (Hsd2^−/−^ 62.66 ± 16.51% versus controls 73.84 ± 16.84%, decreasing to Hsd2^−/−^ 38.39 ± 12.05% versus controls 66.79 ± 13.44%) while intermediate cells staining positive for both aquaporin and V-Atpaseb1 increased during the same period.

### Tissue urea and arginase activity

**Fig. 6:**
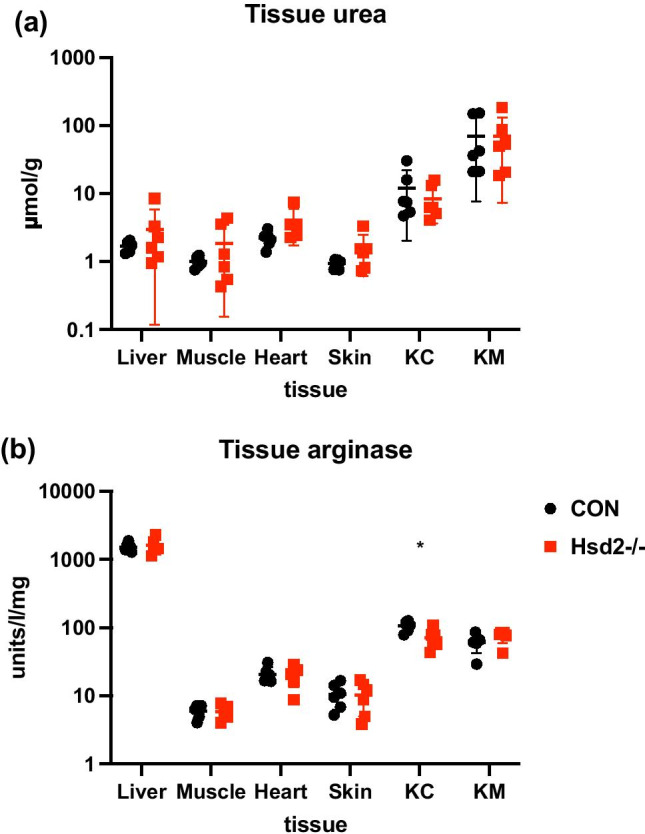
Tissue **a** urea and **b** arginase assays. (KC = kidney cortex; KM = kidney medulla; n = 6 per group; Student’s t test; * < 0.015)

Urea content and arginase activity were determined in adult male liver, muscle, heart, kidney cortex, kidney medulla, and skin samples (Fig. 6a–b). None of the Hsd2^−/−^ tissue samples had significantly different urea content compared to control samples. Arginase activity only reached statistical significance between control and Hsd2^−/−^ kidney cortex samples.

### RNASeq data from adult kidney

Gene expression levels were assessed in adult kidney samples by RNAseq [24]. Key changes in transcripts related to blood pressure and extra cellular volume control are summarized in Table 2. Key changes in electrolyte and water transporter transcripts are summarized in Table 3, and changes in kidney injury and inflammation gene transcripts are given in Table 4. Chronic sodium retention in Hsd2^−/−^ animals was reflected in a 28-fold suppression of renin. Pappalysin 2 was also down-regulated (Pappa2; − 8.28-fold), while the vasopressin receptor was marginally up-regulated (Avpr1a; 1.63-fold). Several Na^+^-transporters were modestly, but significantly, down-regulated (Table 3): the proximal tubule specific Na–H exchanger (Slc9a3; − 1.37-fold) and Na-Pi co-transporter (Slc34a1; − 1.25-fold); the cortical thick ascending limb-specific NKCC2 (Slc12a1; − 1.29-fold), and the distal convoluted tubule-specific NCC (Slc12a3; − 1.36-fold). The water transporters, Aqp1 and Aqp2, were also down-regulated (− 1.31 and − 1.35-fold, respectively). In Hsd2^−/−^ relative to controls, we observed a slight up-regulation in a number of genes specific to the CNT/CCD, including the principal cell-specific αENaC (Scnn1a; 1.66-fold) and the Na^+^/K^+^ transporting subunit alpha 1 ATPase (Atp1a1, 1.33-fold).

**Table 2:** RNAseq data for genes involved in BP and ECV control

	Fold	q value
Ren — renin	− 27.97	0
Pappa2 — pappalysin 2	− 8.28	0
Ptgs2 — Cox2	− 3.1	2.91E-10
Aqp5 — aquaporin 5	− 1.88	6.00E-05
Aqp8 — aquaporin 8	− 1.85	0.0008
Nos1 — nNos	− 1.62	2.40E-09
Agtr1b — angII receptor type 1b	− 1.48	0.005
Aqp2 — aquaporin 2	− 1.35	0.00779
Gcgr — glucagon receptor	− 1.33	6.34E-05
Nr3c2 — mineralocorticoid receptor	− 1.28	0.0013
Aqp1 — aquaporin 1	− 1.25	2.56E-03
Avpr2 — arginine vasopressin receptor 2	− 1.16	1.06E-01
Slc2a2 — Glut2	1.32	1.41E-05
Tgfb1 — transforming growth factor beta 1	1.42	7.61E-05
Edn1 — endothelin 1	1.54	4.21E-05
Avpr1a — arginine vasopressin receptor 1a	1.63	2.88E-06
Cldn7 — claudin 7; membrane protein; Cl permeation	2.01	0

**Table 3:** RNA-seq data for genes coding for nephron transporters

	Fold	q value
Slc14a2 — urea transporter	− 6.4	9.00E-04
Slc22a13 — organic/urate cation transporter	− 3.86	0
Kcna4 — K voltage-gated channel	− 3.45	0
Kcnq3 — K voltage-gated channel	− 2.61	0
Slc4a5 — sodium bicarbonate	− 2.36	0.00015
Slc2a4 — facilitated glucose transfer	− 2.4	0
Atp1a4 — atpase Na/K transporter subunit a4	− 2.13	0
Scn1a — Na voltage-gated channel 1a	− 2.06	0.001
Slc9a3 — Na–H exchanger	− 1.36	0
Slc12a1 — Na K chloride cotransporter (NKCC2)	− 1.29	0.025
Kcnj1 — ROMK channel	− 1.38	0
Slc12a3 — Na-Cl cotransporter (NCC)	− 1.35	0.0016
Slc20a1 — Na dependent Pi cotransporter	1.27	3.00E-05
Car4 — carbonic anhydrase	1.29	0.0008
Atp1a1 — atpase Na/K transporter subunit a1 (IC beta)	1.34	0
Slc4a8 — sodium bicarbonate (IC beta)	1.4	0.0025
Slc4a9 — AE4 (IC β)	1.6	0
Scnn1a — endothelial Sodium chloride (PC)	1.67	0
Kcne5 — K voltage-gated channel	2.03	0.00093
Slc26a4 — Cl/OH/HCo3 exchanger, Pendrin (IC β)	2.04	0
Trpv5 — transient potential cation channel	2.46	0
Kcnma1 — BK, K Ca activated channel, subunit α	2.66	0
Slc34a2 — sodium/phosphate co-transporter	2.68	0
Atp12a — ATPase H/K transporter (distal nephron)	4.33	3.00E-05
Trpv6 — transient potential cation channel	8.89	0

**Table 4:** RNA-seq data for genes involved in fibrosis/inflammation

	Fold	q value
Fgg — fibrinogen gamma	12.62	0
Fgb — fibrinogen beta	9.29	0
C4b — complement component 4b	9.17	0
Spp1 — secreted phosphoprotein	7.86	0
Havcr1 — KIM-1	5.52	0
C4a — complement component 4a	5.16	0.00067
Angptl4 — angiopoietin like 4	4.43	0
Mmp7 — matrix metalloproteinase	4.29	0
Timp1 — tissue inhibitor of metalloproteinase	4.11	0
Fga — fibrinogen alpha	3.97	0
Mmp12 — matrix metalloproteinase	3.18	0
Col1a1 — collagen	2.59	0
Col28a1 — collagen	2.44	0
Fbln1 — fibulin; ECM	2.32	0
Mmp8 — matrix metalloproteinase	2.34	0
Fn1 — fibronectin 1	2.26	0
Mmp2 — matrix metalloproteinase	2.09	0
Col3a1 — collagen	2.08	0
Col8a1 — collagen	2.07	0

The principal cell-specific K^+^ transporter, ROMK, was down-regulated (− 1.39-fold), while the BK alpha sub-unit (Kcnma1), a K^+^ large conductance Ca^2+^-activated channel, which is activated by flow (i.e. polyuria) [24] was significantly up-regulated (2.65-fold), though neither BK beta subunits (Kcnmb1 or Kcnmb4) were altered significantly [19]. Additionally, there was a very significant 4.29-fold up-regulation of the α-intercalated cell-specific HK-ATPase (Atp12a).

A large number of changes associated with kidney damage and extracellular matrix were identified in the adult Hsd2^−/−^ kidney (Table 4), including osteopontin (Spp1; 7.82-fold), Kim-1 (Havcr; 5.52-fold), and Col1a1 (2.58-fold).

## Discussion

The demonstration of high Na^+^ and water content in newborn skin of both SD and F344 rats (despite their demonstrably different handling of Na^+^ [47]) and C57Bl/6 J mice, reveal a species-overarching phenomenon. The high Na^+^ content reflects increased extracellular volume in skin at birth, as has been observed in humans [45]. Oedematous skin at birth may protect the foetus during passage through the birth canal. High skin Na^+^ has been shown to regulate immunity in adult skin [43] so this may represent an innate protection from infection during the transition from an aquatic to dry environment at birth. It has been suggested that Na^+^ is stored in the human foetus and provides a vital source of Na^+^ to support post-natal growth [44]. Additionally, skin may play a role as a ‘rudimentary kidney’, controlling sodium and water balance prior to nephron maturation in the mammalian kidney. It will be important to investigate the sodium status of skin in very premature babies, given the prevalence of trans-epidermal water loss (TEWL) [27, 37].

Using our global rat Hsd11b2 knockout model, we were able to investigate salt handling in very young animals, prior to the development of overt salt-sensitivity. Notably, we found lower Na^+^ and water content in the skin of Hsd2^−/−^ than F344 control newborns. Due to the lack of protective placental Hsd11b2 activity [52], exposure of Hsd2^−/−^ foetuses to maternal glucocorticoids during gestation is likely to affect Na^+^ retention in the skin prior to birth, particularly when the mineralocorticoid receptor is expressed transiently in skin, as has been demonstrated for the mouse at e16 [4]. Boix et al. reported that Hsd11b2 is expressed in mouse skin at e18.5 and that levels diminish post birth [4]. They suggested that Hsd11b2 is expressed in a GR-dependent manner, modulating local glucocorticoid actions. Since pre-term glucocorticoids are known to cause early maturation and increased hydrophobicity of skin [33], presumably through reduction of the extracellular volume, this may explain the reduced Na^+^ and water content in the skin of newborn Hsd2^−/−^ pups.

Salt-induced hypertension in the Dahl salt-sensitive rat has been shown to depend on maternal diet during gestation and lactation, with a casein-based diet exacerbating, and a grain-based diet attenuating salt-sensitivity [16]. The milk recovered from stomachs of Hsd2^−/−^ neonates indicated that Hsd2^−/−^ mothers produce milk with higher [Na^+^] than control dams, which will severely compromise the rapidly growing Hsd2^−/−^ pups. Cross-fostering between control and Hsd2^−/−^ dams would be a way to address this. By 2 weeks of age (when pups are still unable to concentrate urine to the same extent as adults [14]) most Hsd2^−/−^ pups were excreting detectable Na^+^ in the urine, reflecting the increased Na^+^ intake from milk.

During the first week of life Na^+^ and water in the skin (g^−1^ dry weight) declined in both Hsd2^−/−^ pups and controls. During early growth and development, the relative demands on sodium stores may be complex, as demonstrated in the Sprague–Dawley rat [42]. However, the rate of decline in Hsd2^−/−^ pups was half that of controls, and Hsd2^−/−^ pups retained significantly (~ 30%) higher levels of Na^+^ and water in the skin throughout development and into adulthood, indicating an increase in Na^+^ storage and/or reduced Na^+^ clearance from the skin at the expense of K^+^. Beyond birth the increased sodium and water in skin are likely to reflect increased sodium loading — firstly the increased Na^+^ intake from milk and then increased Na^+^ recovery from urine due to the lack of Hsd11b2 activity. Likely consequences of higher skin Na^+^ content include an inability to adapt appropriately to Na^+^ imbalance, by limiting the skin’s capacity to buffer excess Na^+^. It should be noted that the effects seen on skin sodium may reflect increases in total body sodium, as was demonstrated in adult rats, where storage of Na^+^ in skin was highly correlated with skin water, total skin electrolytes, and total body sodium. The exception was bone sodium, which showed a negative correlation with skin Na^+^, both in controls and Hsd2^−/−^ groups. This suggests partitioning of excess Na^+^ in the body, with a completely different control mechanism at play in bone. The apparent paradox between increased Na^+^ recovery in kidney and overt volume contraction (increased haematocrit) may also reflect the partitioning of excess Na^+^ and water.

Incipient hypokalaemia was already evident by 15 days of age. The glucocorticoid surge in the third week post birth exposed the Hsd2^−/−^ pups to additional Na^+^ and water retention through its un-restricted action on the mineralocorticoid receptor in principal cells of the collecting duct, with concomitant K^+^ loss. The overt response post weaning was increasing polydipsia and polyuria, which mirrored the developing hypokalaemia and increased sodium storage. This situation differs from the acute changes seen following administration of a potassium-free diet to adult rats, where polydipsia and polyuria precede overt hypokalaemia [1]. Hypokalaemia has been shown to suppress aquaporin 2 and reduce the capacity of the loop of Henle to generate medullary hypertonicity through the suppression of Slc12a1 and the urea transporter Slc14a2 [13], all of which were down-regulated in the SAME rat. The development of hypokalaemia, in the face of increased Na^+^ partitioning, may set in place irrevocable changes, which result in volume contraction with polydipsia and polyuria as a means to control water balance.

Skin electrolyte concentration remained remarkably constant for both control and Hsd2^−/−^ animals throughout the developmental window observed, suggesting that in Fischer (F344) rats, like the Dahl salt-sensitive rat [47], the electrolytes are associated with water (active) rather than sequestered by proteoglycans in the osmotically inactive form observed in adult DOCA salt rats [46]. Excessive water intake would increase ECV, dropping plasma K^+^ to potentially life-threatening levels, while excessive water loss would decrease ECV, stimulating thirst. We saw no significant changes in urea or arginase activity in tissue samples taken from control or Hsd2^−/−^ adults, indicating that ECV control in this model is not reliant on metabolism-driven natriuretic-ureotelic control [21] but instead depends on tight control of water (and presumably K^+^) balance. The increased urinary Na/K ratio in Hsd2^−/−^ animals reflects a shift in intra/extracellular volume (though this was not tested specifically).

Overt hypertension in Hsd2^−/−^ animals was not seen before 5 weeks of age. It should be noted that all blood pressure measurements were determined under anaesthesia, and though replicate numbers were low because of the technical challenges of working with young neonates, no significant difference was observed between controls and knockouts prior to that age.

RNASeq data from the kidneys of adult Hsd2^−/−^ rats revealed a modest concerted down-regulation of multiple Na^+^ and water transporter mRNAs expressed through the proximal- and mid-nephron. This might occur in response to increased Na^+^ recovery through principal cells, hypokalaemia, and reduced ECV. Though reduction in mRNA levels may not correspond directly to a reduction in active proteins, the large amounts of Na^+^ and urine delivered to the DCT attest to functional down-regulation of transporters in the proximal tubule and the thick ascending limb as has been observed, for example, during angiotensin-converting enzyme inhibition [23]. Captopril, at a dose that does not change BP, has been shown to increase urine flow and depress Na^+^ transport at multiple sites along the nephron, through redistribution or retraction of transporters from the apical membranes in AngII-sensitive regions of the nephron [23]. A similar reduction in transporter abundance and activation also occurs with hypertension [31]. The almost complete suppression of renin in our model indicates that there is very little intra-renal RAS activity so Na^+^ transport in the proximal tubule should be suppressed. Nguyen et al. did not observe many changes in Na^+^, K^+^, and H_2_O transporters along the nephrons of rats fed K^+^-deficient diet in the presence or absence of salt supplementation [32]. In their model, NaCl supplementation caused a reduction in β-ENaC, while NHE3, NKCC2, NCC, Aqp2, and renin were unchanged. Clearly this is significantly different from our model in which ENaC is ‘constitutively’ stimulated and renin highly suppressed.

Up-regulation and stimulation of ENaC activity generates a significant potential driver for K^+^ depletion, which will be exacerbated by inappropriate, flow-induced increase of the BKα channel (2.65-fold increase in mRNA). On the other hand, HK-ATPase (Atp12a), which is stimulated by K^+^ depletion, is a positive K^+^-retaining adaptation to hypokalaemia [9, 53]. The changing proportion of PC to intermediate cells observed beyond weaning may reflect an additional attempt to control or limit Na^+^ uptake, since the activity of ENaC in the intermediate cell is not known. This indicates that the plasticity, observed between principal, intermediate, and intercalated cells, continues up to adulthood in the collecting ducts of rats with SAME.

Interestingly, one of the most significantly down-regulated transcripts was Pappa2 (8.13-fold decrease in mRNA), depletion of which has previously been linked to salt sensitivity in Dahl rats [8]. Pappa2 encodes a metalloproteinase, expressed in the cortical thick ascending limbs of salt-resistant (SR) rats fed a 0.4% sodium diet. It appears to be secreted and subsequently binds to the apical membrane of intercalated cells in the cortical collecting duct. Expression falls dramatically when SR rats are fed an 8% sodium diet, suggesting that its expression is intimately linked to sodium exposure [8]. One of the most significantly up-regulated transcripts was Trpv6 (8.89-fold increase), which has been reported to increase in Gitelman syndrome [57].

The significantly increased percentage of ingested Na^+^ excreted by the adult Hsd2^−/−^ kidney (56% versus 38% in controls) [30] was also observed post weaning, strongly suggesting that the alterations in nephron transporters occurred around weaning, following exposure to incipient hypokalaemia, and compounded by glucocorticoid-stimulated Na^+^ uptake by the principal cell from weaning. The increase of urine flow to the DCT, through down-regulation of Na^+^ transporters and aquaporins in response to hypokalaemia or increased Na^+^ uptake, has been reported to occur within 12 to 24 h [1]. The flexibility of the nephron to adjust expression and trafficking of transporters along its length demonstrates its ability to exquisitely tailor its response to the homeostatic imbalance it is presented with.

There is much debate about the effect of salt on hypertension [48]. Proponents of the Guytonian theory [17] maintain that the kidney is central to regulation of pressure natriuresis [18], while others argue that vascular resistance is key [29]. Data from the kidney-specific Hsd11b2 knockout mouse model [51] suggested that SAME is strictly a kidney phenotype. However, conditional Hsd11b2 knockout in the brain showed that increased salt appetite leads to hypertension [12]. Taken together, evidence from our model of SAME would support the suggestion that the skin also plays a fundamental role in the development of salt-sensitive hypertension.

This is still not the whole story — the Hsd2^−/−^ animals become volume contracted, but paradoxically exhibit oedema of the skin. Despite consuming equivalent amounts of food, adult Hsd2^−/−^ animals are 12% smaller (indicating a catabolic state [28]), have increased insulin sensitivity, and have reduced availability of 11-deoxycorticosteroid for Hsd11b1, in tissues such as the mesenteric fat pad [30]. Both the metabolic dimension (clearly not natriuretic-ureotelic control) and the key roles that potassium and water play in SAME deserve further investigation.

## Experimental procedures

### Experimental animals

All studies were undertaken under UK Home Office license, and ARRIVE guidelines, following review by local ethics committee. Rodents were maintained in a 12-h light–dark cycle (on at 07.00 h) under controlled conditions of humidity (50 ± 10%) and temperature (21 ± 2 °C) and fed rodent maintenance diet (RM1, containing 0.3% Na with soya protein; Special Diet Services Ltd., Witham, Essex, UK) and water ad libitum unless otherwise stated. At end of study or for sample collection, animals were terminated by a schedule 1 method. Hsd11b2 gene was previously knocked out on a Fischer (F344) genetic background, using ZFN gene targeting [30]. Males were used for experimental cohorts, while females were used as breeders for the multiple neonatal groups required.

### Tissue electrolytes

To investigate Na^+^ and water retention in rodent skin at or around birth, skin samples were collected from Sprague–Dawley rats and also from the C57Bl/6J mouse strain at e18.5, newborn, 2 days and 7 days post birth. The smaller, dried mouse skin samples were analysed in Bergen. Wet weight of the samples was measured before being desiccated in a drying chamber, and dry weight was determined when the sample weight was constant. Electrolytes were extracted in 5 ml ultrapure water (Milli-Q, Millipore Corporation) and analysed using highly sensitive and accurate conductivity and charge detection (Thermo Scientific Dionex ICS-4000 System). Based on the measured concentration in the sample, the amount of sodium and potassium relative to the water in the original tissue sample was calculated. Data were compared by two-way ANOVA with non-parametric Kruskal–Wallis and Dunn’s multiple comparison test using Graphpad Prism8 software (p ≤ 0.05 was considered statistically significant).

Skins and pelts were recovered from control Fischer F344 and Hsd11b2^−/−^ newborn, 7-, 13-, and 21-day-old pups and adults (n = 3 to 6 per group). All rat samples were sent to the Experimental & Clinical Research Center (ECRC; Berlin), where they were dried, ashed, and quantified for Na^+^ and K^+^ electrolytes, as previously published [46]. Samples were desiccated at 90 °C for 72 h and water content was calculated. After dry ashing (24 h at 190 °C and 450 °C and finally 600 °C for an additional 48 h) ashes were dissolved in 20 ml 10% HNO3. Electrolyte concentrations were measured with an atomic absorption spectrometer (flame photometry mode; model 3100, PerkinElmer, Rodgau, Germany). Relative changes in electrolytes during growth were calculated according to methods of Shafflhuber et al. [42]. In a similar way, electrolytes were determined in ashed adult carcasses and bones to give an estimate of total body sodium as previously described [46]. Pelt data were compared using two-way ANOVA with Sidak’s multiple comparison test. Newborn skin samples were considered separately, since the newborns experienced a different nutrition experience in utero, compared to older neonates.

### Blood pressure measurement

Male rats were anaesthetized (Inactin, 120 mg/kg ip; n = 3 per age group) and prepared surgically for blood pressure measurements, following catheterization of the carotid and a 40-min equilibration period, using a Powerlab monitor with LabChart software. Blood was collected into heparinized capillary tubes; plasma was separated by centrifugation and the fraction of blood cells (haematocrit; HCT) determined. Data were analysed using Student’s t test.

### Metabolic study

Male control and Hsd2^−/−^ rats (n = 4 per group, 22 to 23 days old) were randomly assigned to and individually housed in metabolic cages (tecniplast) with free access to RM1 and water. Body weight, urine output, food, and water intake were measured daily. Data were analysed by two-way ANOVA with Sidak’s multiple comparison test. Analyses per g body weight from 29 days allowed for acclimatization to the metabolic cage.

### Fluid electrolyte measurements

Plasma [Na^+^] and [K^+^] were determined using the 9180 Electrolyte Analyzer (Roche) (minimum n = 4–6 per time point). Urinary [Na^+^] and [K^+^] were determined using the BWB technologies XP flame photometer. Urine samples (n = 4–6 per time point) were diluted in Bridj detergent buffer (1 in 50 or 1 in 100) and electrolytes measured against standard dilutions. Data were analysed by two-way ANOVA with Sidak’s multiple comparison test.

Stomach contents of neonatal rats were weighed and lyophilized using a vacuum drier (VirTis BenchTop Pro with Omnitronics, SP Scientific, New York; 90mT and – 70 °C for 24 h), extracted in 400 µl of 1 M nitric acid overnight, centrifuged and filtered to remove fine debris. Electrolytes were quantified by flame photometer as above. Flame photometry data were subject to an F-test for normality of variance using StatPlus (AnalystSoft, California). Data were analysed using Student’s t test.

### Tissue urea and arginase activity

Urea and arginase activity were measured as previously published [21]. Briefly, various tissues from adult male F344 control or Hsd2^−/−^ rats (12 weeks old; n = 6) were homogenized in protein extraction reagent (Thermo Fisher Scientific) with an added proteinase inhibitor cocktail (Roche), immediately after tissue collection. Samples were centrifuged at 13,000* g* for 20 min. To extract urea, the samples were centrifuged using a 10-kDa molecular weight cut-off filter (Amicon Ultra, Millipore). The urea-depleted concentrate was used for arginase activity determination, and tissue urea content was measured in the filtrate. Urea concentration was measured in plasma, urine, and tissue using a BioVision urea assay kit. Tissue arginase activity was measured using an arginase assay kit (Sigma-Aldrich). Data were analysed using the Student t test.

### Principal cell (PC): intercalated cell (IC): intermediate cell ratios during development

Kidneys were dissected from male rats aged 3 to 5 weeks and 6 months and fixed overnight in 4% paraformaldehyde at 4 °C. Following paraffin embedding, 5µ sections were processed by de-waxing, rehydration, and heat induced antigen retrieval in sodium citrate buffer pH6. Double immunostaining was carried out using polyclonal goat anti-mouse Aqp2 (NovusBio NBP1-70,378, 1:1000 for mouse and 1:500 for rat) and polyclonal rabbit anti-human V-ATPase B1 (1:200 for mouse and 1:50 for rat). The secondary antibodies used were polyclonal donkey anti-goat AlexaFluor 488 (Life Technologies, A-11055) and donkey anti-rabbit AlexaFluor 568 (Life Technologies, A10042). Stained samples were imaged using a Q-imaging camera (Canada) on a Nikon Eclipse Ti fluorescent microscope with DAPI, FITC and TRITC filters applied, for DAPI, AlexaFluor 488, and AlexaFluor 568, respectively. ﻿Both 60X 1.4 NA Plan Apo and 40X 1.3 NA Plan Flur oil objectives were used. A minimum of 6 images for each section were analysed using ImageJ software (National Institutes for Health). The cell counter was blinded to the source of each section. Cells within collecting ducts expressing Aqp2 only were deemed principal cells, those expressing V-ATPase B1 as intercalated cells and those expressing both as an intermediate cell type. The relative proportion of cell types in collecting ducts were counted in control and Hsd2^−/−^ rats at ~ 5 weeks (n = 4) and 24 weeks (n = 3). The log-ratio of PC: IC and intermediate: IC are reported as means ± SD. Significance between groups was assessed using MANOVA test with p < 0.05 (*). A decrease in the log-ratio of PC:IC indicates an overall decrease in the proportion of PCs present in the CD relative to ICs, which did not significantly change between WT and KO. Likewise, an increase in the log-ratio of intermediate: IC indicates an overall increase in the proportion of intermediate cells relative to ICs.

### RNASeq

RNA was prepared from control and Hsd2^−/−^ adult male whole kidney samples (23–25 weeks of age; n = 6 per group) using trizol and quality assessed by bio-analyzer. RNA-seq data from a Tru-Seq stranded library was obtained on a NextSeq 550 with 75 bp reads and was quantified and analysed for differential expression and over-representation of gene sets. Quality checks were made with FASTQC [Reference Source (2010)] and trimming was applied using Trimmomatic [5]. Transcript quantification was carried out with quasi-alignment using Salmon [34], and reads were aligned with HISAT2 [20] to the Ensembl rat Rnor_6.0 genome (rn6). Stranded-ness and distribution of reads by genomic feature was assessed using infer_experiments.py and read_distribution.py from RSeQC [20, 54] following an initial un-stranded alignment, with > 98% of reads exhibiting the expected stranded-ness. Estimated counts adjusted for library size and transcript length were derived from the Salmon results with tximport, normalization with the trimmed mean of M values [40], differential expression analysis with edgeR version 3.12.0 [39], and differential exon usage analysis with DEXSeq version 1.16.6 [25]. Canonical pathways, hallmarks, KEGG, and gene ontology libraries were downloaded from version 5.0 of the molecular signatures database [26]. Identifiers for all gene sets were mapped to rat via homology relationships downloaded from the RGD database (RGD_ORTHOLOGS.txt) [6]. Differences in gene set expression between experimental groups were examined via the statistically robust ROAST method [56], as implemented in the limma package of Bioconductor (version 3.27.4) [38].

### Statistical analysis

Statistical analyses were chosen according to the design of each experiment (Student’s t test, one-way ANOVA, or two-way ANOVA) using Graphpad Prism8 software or multiple analysis of variance using R. All graphs are presented ± SD.

## Data Availability

RNAseq data is lodged with ArrayExpress (Accession E-MTAB-10478).
